# Supplementary material for: The Use of Glucagon-like Peptide-1 Receptor Agonists in Patients with Type 2 Diabetes Mellitus Does Not Increase the Risk of Pancreatic Cancer: A U.S.-Based Cohort Study
Source: Cancers (Basel). 2024 Apr 23;16(9):1625. doi: 10.3390/cancers16091625 (PMC11082986; doi:10.3390/cancers16091625)
Supplement: Supplementary file 1 [file cancers-16-01625-s001.zip › cancers-2965518-supplementary.pdf]

Supplementary Material: ICD-10 and Identification Codes Used in Methodology

• ***Patient Diagnoses***		
T2DM	Type 2 diabetes mellitus	ICD-10 E11
Personal Carrier	Genetic carrier of other disease	ICD-10 Z15.09
	Genetic susceptibility to other malignant neoplasm	ICD-10 Z14.8
Genome	NOTCH1	GENE 7881
Genome	BRCA1	GENE:1100
Genome	BRCA2	GENE:1101
Genome	CDKN2A	GENE:1787
Genome	KRAS	TNX:386601
Genome	MEN1	GENE:7010
Genome	MLH1	GENE:7127|var
Genome	MSH2	GENE:7325|var
Genome	NOTCH1	GENE:7881
Genome	PALB2	GENE:26144
Genome	PMS2	GENE:9122
Genome	PRSS1S	LNC:21692-9
Genome	VHL	GENE:12687
Pancreatic Cyst	Cyst of pancreas	ICD-10 K86.2
Family History	Family history of malignant neoplasm of digestive organs	ICD-10 Z80.0
Family Carrier	Family history of carrier of genetic disease	ICD-10 Z84.81
• ***Outcomes***		
Pancreatic Cancer	Malignant neoplasm of pancreas	ICD-10 C25

ICD-10: International Classification of Diseases 10th Revision; T2DM: Type 2 Diabetes Mellitus
